# Hypoxia Induces Macrophage *tnfa* Expression via Cyclooxygenase and Prostaglandin E2 *in vivo*

**DOI:** 10.3389/fimmu.2019.02321

**Published:** 2019-09-27

**Authors:** Amy Lewis, Philip M. Elks

**Affiliations:** The Bateson Centre and Department of Infection, Immunity and Cardiovascular Disease, University of Sheffield, Sheffield, United Kingdom

**Keywords:** hypoxia, TNF–tumour necrosis factor, COX–cyclooxygenase, zebrafish, prostaglandin, macrophage, *in vivo*, HIF–hypoxia inducible factor

## Abstract

Macrophage phenotypes are poorly characterized in disease systems *in vivo*. Appropriate macrophage activation requires complex coordination of local microenvironmental cues and cytokine signaling. If the molecular mechanisms underpinning macrophage activation were better understood, macrophages could be pharmacologically tuned during disease situations. Here, using zebrafish *tnfa:GFP* transgenic lines as *in vivo* readouts, we show that physiological hypoxia and stabilization of Hif-1α promotes macrophage *tnfa* expression. We demonstrate a new mechanism of Hif-1α-induced macrophage *tnfa* expression via a cyclooxygenase/prostaglandin E2 axis. These findings uncover a macrophage HIF/COX/TNF axis that links microenvironmental cues to macrophage phenotype, with important implications during inflammation, infection, and cancer, where hypoxia is a common microenvironmental feature and where cyclooxygenase and TNF are major mechanistic players.

## Introduction

Innate immune activation during homeostasis and disease are tightly regulated, in part by the coordination of microenvironmental cues and cytokine signaling. Macrophages are important innate immune cells in disease and their activation status, commonly termed polarization, is especially important during the response to injury and infection. Mammalian macrophages have been classified into two broad polarization states: M1 (or classically activated) and M2 (alternatively activated) (1, 2). M1 macrophages are highly antimicrobial and can phagocytose and efficiently kill bacteria. M2 macrophages are central players in tissue healing and restoration of homeostasis post injury/infection (3, 4). M1/M2 polarization states have largely been determined *in vitro* by addition of exogenous factors, e.g., cytokines, to the growth media, however the complex regulation of the diverse macrophage phenotypes observed *in vivo* remains poorly understood. It is only in whole organism *in vivo* models, where tissues remain intact, that microenvironmental cues of macrophage activation can be fully investigated.

Leukocytes, including macrophages and neutrophils, are exquisitely sensitive to tissue hypoxia, a signature of many disease microenvironments (e.g., in inflammatory diseases, infections, and cancers) due to lack of local blood supply and a high turnover of local oxygen by pathogens (5, 6). Tissue hypoxia can polarize macrophages, via stabilization of the Hypoxia Inducible Factor (HIF)-1α transcription factor (7). HIF-1α is a master transcriptional regulator of the cellular response to hypoxia (8) and has been shown to have activating effects on macrophages *in vitro*, increasing their pro-inflammatory cytokine profile and bactericidal capabilities, yet the molecular mechanisms behind these observations are not well-understood *in vivo* (6).

Eicosanoids are lipid signaling molecules and important pro-inflammatory mediators during homeostasis and disease (9, 10). For example, tissue hypoxia increases cyclooxygenase (COX)/prostaglandin E2 (PGE2) production in epithelial cells in cancer models (11–13). Recent evidence indicates that hypoxia-induced tumor necrosis factor alpha (TNF) expression in osteoblasts (bone producing cells) is mediated through cyclooxygenase enzymes by an, as yet, unknown mechanism (14). This HIF/COX/TNF axis has not, to date, been observed in macrophages, and could be a potential novel pathway to macrophage activation in diseases where hypoxia is a microenvironmental factor.

Here, we investigated the regulation of expression of the important, primarily pro-inflammatory, cytokine *tnfa* by Hif-1α stabilization *in vivo* using optically transparent zebrafish larvae. We demonstrate that genetic, pharmacological, and hypoxia-mediated Hif-1α stabilization upregulated macrophage *tnfa*. We utilize well-characterized, non-invasive, zebrafish models of inflammation and infection to show that Hif-1α-mediated *tnfa* acts via an alternative, cyclooxygenase dependent mechanism, that differs from better understood DAMP/PAMP mediated pathways that are cyclooxygenase independent (15, 16). We show that the Hif-1α-mediated *tnfa* acts via active PGE2. Furthermore, this new macrophage HIF/COX/TNF axis is found in primary human macrophages. These findings have important implications in inflammation, infection, and cancer biology where macrophage phenotypes are influenced by microenvironmental hypoxia, and where HIF, COX, and TNF are major mechanistic players.

## Materials and Methods

### Zebrafish

Zebrafish were raised and maintained on a 14:10 h light/dark cycle at 28°C, according to standard protocols (17), in UK Home Office approved facilities at The Bateson Center aquaria at the University of Sheffield. Strains used were Nacre (wildtype), *TgBAC(tnfa:GFP)pd1028* (Source: Bagnat lab, Duke University, USA), *Tg(tnfa:eGFP-F)ump5Tg:Tg(mpeg1:mCherry-F)ump2Tg* (Source: Lutfalla Lab, Montpellier University, France), *Tg(mpeg1:mCherryCAAX)sh378* (Source: University of Sheffield)*, Tg(lyz:Ds-RED2)nz50* (Source: Hall Lab, University of Auckland, New Zealand), *TgBAC(il-1*β*:eGFP)sh445* (Source: University of Sheffield), and *Tg(phd3:EGFP)i144* (Source: University of Sheffield) (18–22).

### Tailfin Injury

Inflammation was induced in zebrafish embryos by tail transection at 2 days post-fertilization (dpf). Larvae were anesthetized and the tailfin was transected using a sterile scalpel blade (23). Larvae were imaged by confocal microscopy at 16 h post-wounding (hpw) on a Leica TCS-SPE confocal on an inverted Leica DMi8 base and imaged using 20× or 40× objective lenses.

### Mycobacterium marinum

*Mycobacterium marinum* (Mm) M (ATCC #BAA-535), containing a psMT3-mCherry or psMT3 mCrimson vector were used (24). Injection inoculum was prepared from an overnight liquid culture in the log-phase of growth resuspended in 2% polyvinylpyrrolidone40 (PVP40) solution (CalBiochem, CAS 9003-39-8) (25). One hundred or one hundred and fifty colony forming units (CFU) were injected into the caudal vein at 28–30 h post-fertilization (hpf) (26).

### Confocal Microscopy of Transgenic Larvae

Larvae were mounted in 0.8–1% low melting point agarose (Sigma-Aldrich, A9414) and imaged on a Leica TCS-SPE confocal on an inverted Leica DMi8 base and imaged using 20× or 40× objective lenses.

For quantification purposes acquisition settings and area of imaging were kept the same across groups. Corrected total cell fluorescence was calculated for each cell using Image J by drawing around the cell and correcting for background levels of staining (27, 28).

### RNA Injections

Embryos were injected with dominant *hif-1*α*b* (ZFIN: *hif1ab*) variant RNA at the one cell stage as previously described (23). *hif-1*α variants used were dominant active (DA) that is a constitutively stable Hif-1α and dominant negative (DN) *hif-1*α that cannot signal due to lack of transactivation domain. Phenol red (PR) (Sigma-Aldrich, P0290) was used as a vehicle control.

### Bacterial Pixel Count

Mm mCherry infected zebrafish larvae were imaged at 4 days post-fertilization (dpi) on an inverted Leica DMi8 with a 2.5× objective lens. Brightfield and fluorescent images were captured using a Hammamatsu OrcaV4 camera. Bacterial burden was assessed using dedicated pixel counting software as previously described (29).

### Pathway Inhibitors

Unless otherwise stated, embryos were treated from 4 h pre-Mm infection to 24 h post-infection (hpi) by addition to the embryo water in six well plates and dimethyl sulfoxide (DMSO, Sigma-Aldrich, D8418), was used as a negative solvent control. The pan hydroxylase inhibitor, DMOG (dimethyloxaloylglycine, Enzo Life Sciences, BML-EI347-0010), was used at a 100 μM concentration by incubation in E3 embryo media (23). The selective PHD inhibitors JNJ-402041935 (Cayman Chemicals, 14316) and FG-4592 (Selleckchem, FG-34595) were used at 100 and 5 μM, respectively (30, 31). The selective inhibitor of COX-1, SC-560 (Cayman Chemical, 70340), was used at 30 μM and the selective inhibitor of COX-2, NS-398 (Cayman Chemical, 70590), was used at 15 μM by incubation in E3 embryo media (32). The 15-LOX inhibitor PD146176 (Tocris, 2850) was microinjected (1 nl of 40 nM) at 1 h post-infection (hpi) (33). The leukotriene B4 receptor 1 (BLTR1) inhibitor, U-75302 (Abcam, ab141736), was used at 30 mM by incubation in E3 embryo media (33) together with the BLTR2 inhibitor, LY255283 (Abcam, ab144472), which was used at 1 mM (34). Exogenous prostaglandin E2 (Cayman Chemical, 14010) and 15-keto-prostaglandin E2 (Cayman Chemical, 14720) were added by incubation in E3 at a concentration of 1 μM (35). The EP4 antagonist AH23848 (Cayman Chemical, 19023) was used at 1 μM (36).

### Hypoxia Hood

Embryos were incubated in 5% oxygen (with 5% carbon dioxide) in a hypoxia hood (Baker-Ruskinn Sci-tive UM-027) from 32 hpi for 6 h and were imaged at 2 dpf. Embryos from the same clutch kept in incubated normoxic room air were used as controls (37).

### Human Cells

Peripheral blood mononuclear cells (PBMC) were isolated by Ficoll-Paque PLUS (GE Healthcare, GE17-1440-03) density centrifugation of whole blood from healthy donors (National Research Ethics Service reference 07/Q2305/7). PBMC were seeded in RMPI 1640 media (Gibco, 11875-093) containing 2 mmol/L L-glutamine (Lonza, BE17-605F) and 10% newborn calf serum (Gibco, 26010074). Cells were cultured in 5% CO_2_ at 37°C for 14 days in RPMI 1640 supplemented with 2 mmol/L L-glutamine and 10% heat-inactivated fetal bovine serum (FBS) (PAN Biotech, P30-3306) (38).

RPMI 1640 + 25 mM HEPES (Gibco, 11550496) containing 2 mmol/L L-glutamine and 10% heat-inactivated FBS and drugs were pre-equilibrated in normoxia or hypoxia (0.8% O_2_, 5% CO_2_, 70% humidity at 37°C, in a Baker-Ruskinn Sci-Tive UM-027) for 24 h prior to the experiment (39). Cells were treated in duplicate or triplicate wells with 100 μg/ml LPS (InvivoGen, tlrl-b5lps), DMSO (Sigma-Aldrich, D8418), or 15 mM COX-2 inhibitor (NS398) (Cayman Chemical, 70590) and incubated for 18 h. For normoxic controls, cells were treated identically within a class-2 tissue culture hood and transferred to a normoxic tissue-culture incubator.

Cell supernatants were collected following 18 h incubation and were assayed in triplicate using a human TNF ELISA MAX (Biolegend, 430204).

### Statistical Analysis

All data were analyzed (Prism 7.0, GraphPad Software) using unpaired, two-tailed *t*-tests for comparisons between two groups and ANOVA (with Bonferonni post-test adjustment) for other data. *P*-values shown are: *P < 0.05, **P < 0.01, and ***P < 0.001.

## Results

### Hypoxia and Hif-1α Stabilization Upregulate *tnfa:GFP* Expression *in vivo*

**Figure 1:**
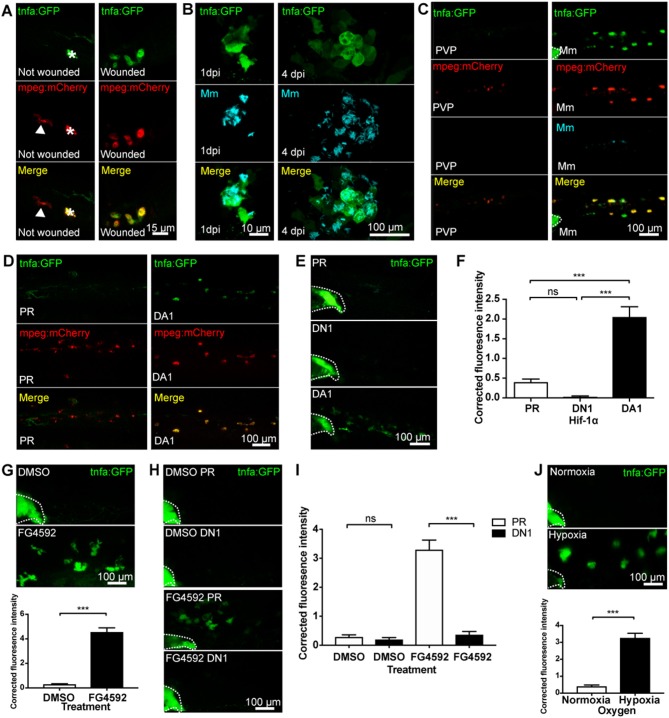
Macrophage *tnfa:GFP* is upregulated by injury, Mm infection and Hif-1α stabilization. **(A)** Confocal micrographs of 3 days post-fertilization larvae with or without tailfin wound (16 hpw) at the tailfin site. *tnfa* expression was detected using the *TgBAC(tnfa:GFP)pd1028* transgenic line. Macrophages are shown in red using a *Tg(mpeg1:mCherryCAAX)sh378* line. The white arrowhead indicates the presence of a *tnfa:GFP* negative, *mpeg:mCherry* positive macrophage in the tailfin of a non-wounded control. Asterisk indicates a neuromast that is fluorescent in all channels as a marker of position. Representative images of three independent experiments with 20–30 larvae per group per experiment. **(B)** Confocal micrographs of 1 dpi Mm mCrimson infected larvae, prior to granuloma formation (left panels), and 4 dpi, early granuloma (right panels) stages of infection using the *TgBAC(tnfa:GFP)pd1028* line. Representative images of three independent experiments with 20–30 larvae per group per experiment. **(C)** Confocal micrographs of 1 day post-infection (dpi) with Mm mCrimson at the caudal vein region of infection in the *TgBAC(tnfa:GFP)pd1028* line. Macrophages are shown in red using a *Tg(mpeg1:mCherryCAAX)sh378* line. Mm in right panels with PVP control in left panels. a Dotted lines indicate the yolk extension of the larvae where there is non-specific fluorescence. Representative images of three independent experiments with 20–30 larvae per group per experiment. **(D)** Confocal micrographs of 2 dpf larvae in the caudal vein region in the *TgBAC(tnfa:GFP)pd1028* line. Macrophages are shown in red using a *Tg(mpeg1:mCherryCAAX)sh378* line. Larvae were injected at the 1 cell stage with dominant active (DA) Hif-1α or phenol red (PR) control. Representative images of three independent experiments with 20–30 larvae per group per experiment. **(E)** Confocal micrographs of 2 dpf zebrafish imaged around the caudal vein region in the *TgBAC(tnfa:GFP)pd1028* line. Larvae were injected at the 1 cell stage with dominant negative (DN) or dominant active (DA) Hif-1α or phenol red (PR) control. **(F)** Corrected fluorescence intensity levels of *tnfa:GFP* in larvae in **(E)**. Mean ± SEM, *n* = 66 cells from 12 embryos accumulated from three independent experiments and corresponds to images in **(E)**. P-values shown are: *P < 0.05, **P < 0.01, and ***P < 0.001, one way ANOVA with Bonferonni post-test adjustment. **(G)** Confocal micrographs of 2 dpf *TgBAC(tnfa:GFP)pd1028* larvae treated with the PHD inhibitor FG4592 or a DMSO solvent control. Graph shows corrected fluorescence intensity levels of *tnfa:GFP*. Mean ± SEM, *n* = 54 cells from nine embryos accumulated from three independent experiments. *P*-values shown are: *P < 0.05, **P < 0.01, and ***P < 0.001, unpaired, two-tailed *t*-test. **(H)** Confocal micrographs of 2 dpf *TgBAC(tnfa:GFP)pd1028* larvae treated with the PHD inhibitor FG4592 or a DMSO solvent control. Larvae were injected at the 1 cell stage with dominant negative (DN) Hif-1α or phenol red (PR) control. **(I)** Corrected fluorescence intensity levels of *tnfa:GFP* in larvae in **(H)**. Mean ± SEM, *n* = 60 cells from 10 embryos accumulated from three independent experiments. *P*-values shown are: *P < 0.05, **P < 0.01, and ***P < 0.001, two way ANOVA with Bonferonni post-test adjustment. **(J)** Confocal micrographs of 2 dpf *TgBAC(tnfa:GFP)pd1028* larvae incubated in room normoxia or 5% oxygen (hypoxia) for 6 h between 32 and 38 hpf. Graph shows corrected fluorescence intensity levels of *tnfa:GFP*. Mean ± SEM, *n* = 72 cells from 12 embryos accumulated from three independent experiments. *P*-values shown are: *P < 0.05, **P < 0.01, and ***P < 0.001, unpaired, two-tailed *t*-test.

Tumor necrosis factor (TNF) is a central regulator of macrophage pro-inflammatory phenotypes during homeostasis, inflammation, and infection. Here, we use two transgenic zebrafish lines that express GFP under the control of a *tnfa* promoter region; *TgBAC(tnfa:GFP)pd1028* and *Tg(tnfa:eGFP-F)ump5Tg* (18, 20). The *TgBAC(tnfa:GFP)pd1028* is a BAC (bacterial artificial chromosome) transgenic line that contains 50 kb of promoter region (18). The *Tg(tnfa:eGFP-F)ump5Tg* line has a smaller promoter region (3.8 kb) than the BAC line (20) and has previously been demonstrated to be upregulated in macrophages after tailfin injury in a zebrafish model (20). Here, we demonstrate that injury-induced upregulation of macrophage *tnfa:GFP* is also found in the BAC transgenic line at 16 h post-wound (hpw), shown in double transgenic *TgBAC(tnfa:GFP)pd1028; Tg(mpeg1:mCherryCAAX)sh378* larvae (Figure 1A).

TNF has been implicated in many bacterial infections, however the cell types involved have been difficult to observe *in vivo*. To investigate this we used a well-characterized zebrafish infection model, the *M. marinum* (Mm) model of tuberculosis infection (40). We used the *tnfa* BAC promoter GFP line to establish the transcriptional regulation of *tnfa* during pre- and early- larval Mm granuloma formation stages. *tnfa:GFP* expression was induced in larvae following Mm infection before granuloma onset (at 1 day post-infection, dpi), and during granuloma formation (at 4 dpi) (Figure 1B). *tnfa:GFP* expression was predominantly found in macrophages, shown by co-localization of fluorescence with *mpeg-1:mCherry* expressing macrophages (Figure 1C). Macrophage expression was confirmed using the other *tnfa* promoter driven line, *Tg(tnfa:eGFP-F)ump5Tg*; *Tg(mpeg1:mCherry-F)ump2Tg* (Figure S1). Our data demonstrate that injury and Mm induced *tnfa* expression occurs in macrophages as part of an early response.

Hypoxia and Hif-1α stabilization are key features of diseased tissue microenvironments that can trigger macrophage pro-inflammatory responses. We first tested whether Hif-1α promotes *tnfa in vivo*, by stabilizing Hif-1α genetically in the *tnfa:GFP* BAC transgenic line (18, 20). Dominant active (DA) Hif-1α RNA (23) resulted in upregulation of macrophage *tnfa:GFP* expression (Figures 1D–F) indicating a shift of macrophage phenotype toward a pro-inflammatory response. Dominant negative (DN) Hif-1α caused no change in *tnfa:GFP* expression (Figures 1E,F).

To ensure that endogenous levels of Hif-1α could trigger a *tnfa* response, Hif-α was stabilized in embryos pharmacologically. Treatment with the well-described hypoxia mimetic DMOG (dimethyloxaloylglycine, a pan-hydroxylase inhibitor that stabilizes endogenous Hif-1α via inactivation of regulatory prolyl hydroxylase enzymes) alongside newer hydroxylase inhibitors with reportedly greater prolyl hydroxylase selectively than DMOG, JNJ-42041935, and FG4592 were tested in the *Tg(phd3:GFP)i144* hypoxia reporter zebrafish. *phd3* is a Hif-1α transcriptional target that is highly upregulated after hypoxia/Hif-1α stabilization (22). Treatment with all the hydroxylase inhibitors increased *phd3:GFP* levels compared to DMSO controls demonstrating stabilization of the Hif-α pathway (Figure S2) with FG4592 treatment leading to the highest *phd3:GFP* levels (23, 30, 31, 37). FG4592 (Figure 1G), DMOG (Figure S3A), and JNJ-42041935 (Figure S3B) treatment upregulated *tnfa*:*GFP* expression compared to a DMSO solvent control, phenocopying the DA Hif-1α response. *tnfa:GFP* induced by hydroxylase inhibition could be blocked using dominant negative (DN) Hif-1α, both in the case of FG4592 (Figures 1H,I) and DMOG (Figure S4), demonstrating that hydroxylase inhibitors are acting via Hif-1α to induce *tnfa:GFP*.

Finally, we subjected the *tnfa:GFP* line to physiological hypoxia (5% oxygen for 6 h at 32 hpf) and looked for GFP expression at 48 hpf. This level of hypoxia was sufficient to turn *on phd3:GFP* expression in *Tg(phd3:GFP)i144* hypoxia reporter zebrafish (Figure S5) (37). Hypoxia induced *tnfa:GFP* expression, compared to normoxic controls at 48 hpf, to a similar level as that observed with genetic or pharmacological Hif-1α stabilization (Figure 1J).

Together, these data indicate that *tnfa* expression is induced in macrophages in response to hypoxia and Hif-1α, a response that is targetable by pharmacological agents and has the potential to manipulate macrophage responses during disease.

### Hif-1α Dependent *tnfa:GFP* Transcription Requires Cyclooxygenase

**Figure 2:**
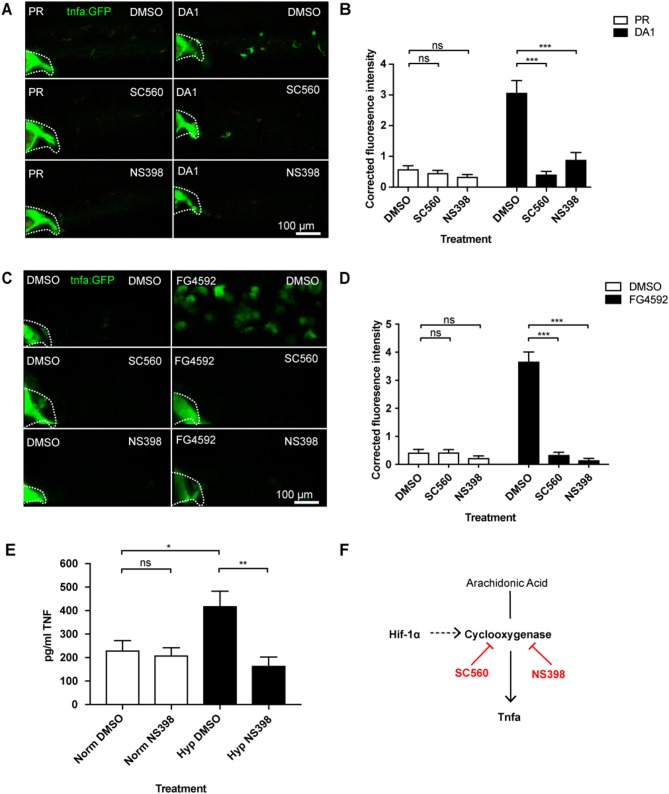
Hif-1α-activated *tnfa* is cyclooxygenase dependent. **(A)** Confocal micrographs of 2 dpf caudal vein region of larvae in the *TgBAC(tnfa:GFP)pd1028* line. Phenol red (PR) and dominant active Hif-1α (DA1) injected larvae were treated with DMSO, SC560 (Cox-1 inhibitor), and NS398 (Cox-2 inhibitor). Dotted lines indicate the yolk extension of the larvae where there is non-specific fluorescence. **(B)** Corrected fluorescence intensity levels of *tnfa:GFP* in larvae in **(A)**. Mean ± SEM, *n* = 36 cells from six embryos representative of three independent experiments. *P*-values shown are: *P < 0.05, **P < 0.01, and ***P < 0.001, two way ANOVA with Bonferonni post-test adjustment. **(C)** Confocal micrographs of 2 dpf caudal vein region of larvae in the *TgBAC(tnfa:GFP)pd1028* line. DMSO and FG4592 treated larvae were co-treated with DMSO, SC560 (Cox-1 inhibitor), and NS398 (Cox-2 inhibitor). Dotted lines indicate the yolk extension of the larvae where there is non-specific fluorescence. **(D)** Corrected fluorescence intensity levels of *tnfa:GFP* in larvae in **(C)**. Mean ± SEM, *n* = 48 cells from eight embryos representative of three independent experiments. *P*-values shown are: *P < 0.05, **P < 0.01, and ***P < 0.001, two way ANOVA with Bonferonni post-test adjustment. **(E)** TNF ELISA of human monocyte derived macrophages treated with LPS and incubated in normoxia or 0.8% hypoxia with or without treatment with NS398. LPS negative controls were performed but TNF produced in these groups was below detectable levels. Mean ± SEM, *n* = 5–8 biological repeats from 3 to 4 donors. *P*-values shown are: *P < 0.05, **P < 0.01, and ***P < 0.001, two way ANOVA with Bonferonni post-test adjustment. **(F)** Schematic of the arachidonic pathway indicating that stabilizing Hif-1α upregulates *tnfa*, an effect that is blocked using the Cox1/2 inhibitors SC560/NS398.

Eicosanoids are lipid signaling molecules and can be produced by macrophages as part of a pro-inflammatory response (9, 10). We tested whether cyclooxygenase inhibition affected Hif-1α-mediated macrophage *tnfa:GFP* expression, using Cox-1 and Cox-2 inhibitors (SC560 and NS398, respectively) (32). First, we showed that neither SC560 nor NS398 caused increased Hif-α transcriptional activity, using the *Tg(phd3:GFP)i144* hypoxia reporter zebrafish (Figure S6). In the absence of Hif-1α stabilization *tnfa:GFP* was not altered by SC560 or NS398 compared to DMSO treated negative controls (Figures 2A,B). Strikingly, the DA Hif-1α-induced *tnfa:GFP* expression was reduced to basal levels by both SC560 and NS398 (Figures 2A,B). These findings were replicated using the hydroxylase inhibitors FG4592 (Figures 2C,D) and DMOG with NS398 (Figure S7), (N.B. DMOG with SC560 caused toxicity and developmental delay therefore data is not shown for this group). The production of an unrelated Hif-1α target gene *phd-3* by hydroxylase inhibitors, shown in the *Tg(phd3:GFP)i144* transgenic line, was not altered by SC560 or NS398 treatment, demonstrating that Hif-1α transcriptional potential is not affected by cyclooxygenase inhibition (Figure S8).

We tested whether HIF-1α stabilization induced TNF in human monocyte derived macrophages (hMDMs). We found that human monocyte derived macrophages produced higher levels of TNF protein in hypoxia (0.8% oxygen) than those in normoxia, measured by an anti-human TNF ELISA (Figure 2E). Furthermore, treatment with the COX-2 inhibitor, NS398, reduced this hypoxia-induced TNF to equivalent levels found in normoxia (Figure 2E). This was replicated when HIF-1α was stabilized in hMDMs using the hypoxia mimetic FG4592 (Figure S9). These data indicate that a HIF/COX/TNF axis is found in zebrafish and human macrophages, and could be a druggable target in disease situations (Figure 2F).

We have previously shown that DA Hif-1α induces the expression of another important pro-inflammatory cytokine, *il-1*β, shown by an *il-1*β*:GFP* transgenic line (Figure S10A) (37). Cox-1 inhibition by SC560 did not abrogate Hif-1α-induced *il-1*β*:GFP*, while Cox-2 inhibition with NS398 caused a small decrease that did not reach basal levels (Figures S10B,C). These data indicate that Hif-1α-induced *tnfa* expression is caused by a product of the cyclooxygenase arm of the arachidonic acid pathway via a novel macrophage HIF/COX/TNF axis, while Hif-1α-induced *il-1*β does not fully act via this pathway suggestive of complex regulation of inflammatory cytokines by Hif-1α stabilization.

**Figure 3:**
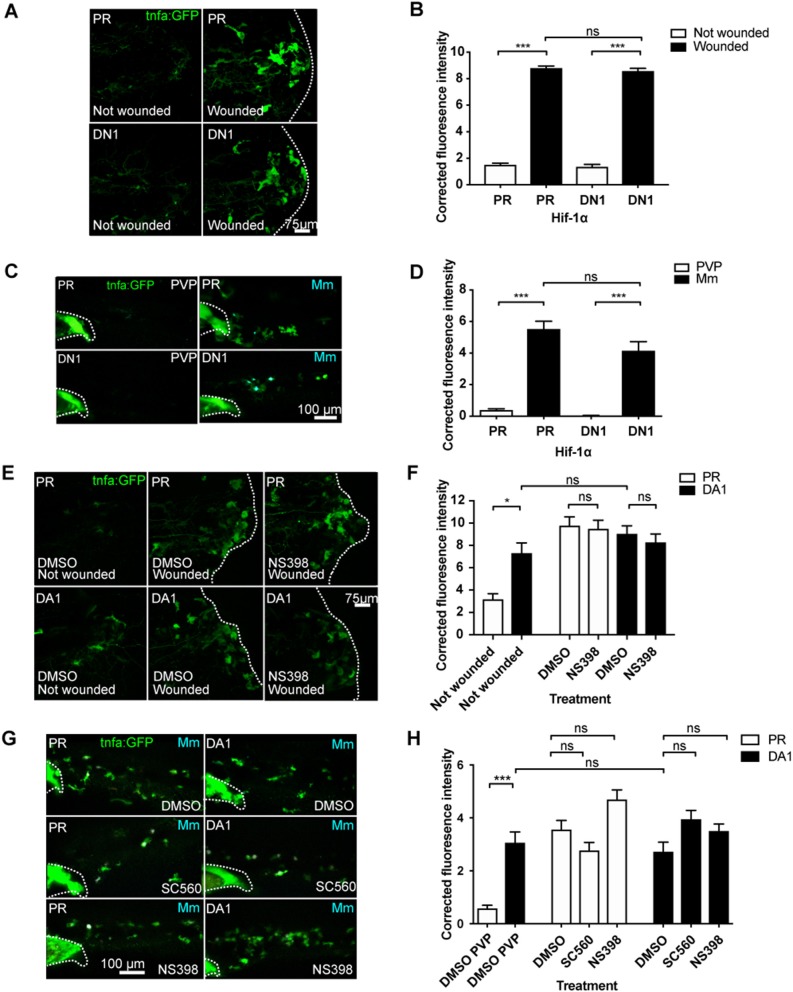
Injury and infection induced *tnfa:GFP* is cyclooxygenase independent. **(A)** Confocal micrographs of the *TgBAC(tnf*α*:GFP)pd1028* line at 3 days post-fertilization larvae with or without tailfin wound (induced 16 h previously) in phenol red (PR) or dominant negative (DN) Hif-1α embryos. The dotted line indicates the border of the tailfin wound. **(B)** Corrected fluorescence intensity levels of *tnfa:GFP* in larvae in **(A)**. Mean ± SEM, *n* = 60 cells from 10 embryos accumulated from three independent experiments. *P*-values shown are: *P < 0.05, **P < 0.01, and ***P < 0.001, two way ANOVA with Bonferonni post-test adjustment. **(C)** Confocal micrographs of 1 dpi caudal vein region of larvae in the *TgBAC(tnfa:GFP)pd1028* line. Phenol red (PR) and dominant negative Hif-1α (DN1) injected larvae were infected with Mm Crimson or PVP controls. **(D)** Corrected fluorescence intensity levels of *tnfa:GFP* in larvae in **(C)**. Mean ± SEM, *n* = 30 cells from five embryos accumulated from two independent experiments. *P*-values shown are: *P < 0.05, **P < 0.01, and ***P < 0.001, two way ANOVA with Bonferonni post-test adjustment. **(E)** Confocal micrographs of the *TgBAC(tnf*α*:GFP)pd1028* line at 3 days post fertilization larvae with or without tailfin wound (induced 16 h previously) in phenol red (PR) or dominant active Hif-1α embryos. Embryos were treated with DMSO or NS398 (Cox-2 inhibitor). **(F)** Corrected fluorescence intensity levels of *tnfa:GFP* in larvae in **(E)**. Mean ± SEM, *n* = 24 (in not wounded) or *n* = 36 (in wounded) cells from six embryos accumulated from two independent experiments. n.b. Not-wounded fish had fewer macrophages at the tailfin site. *P*-values shown are: *P < 0.05, **P < 0.01, and ***P < 0.001, two way ANOVA with Bonferonni post-test adjustment. **(G)** Confocal micrographs of 1 dpi caudal vein region of Mm Crimson infected larvae in the *TgBAC(tnfa:GFP)pd1028* line. Phenol red (PR) and dominant active Hif-1α (DA1) injected larvae were treated with DMSO, SC560 (Cox-1 inhibitor), and NS398 (Cox-2 inhibitor) and infected with Mm Crimson. **(H)** Corrected fluorescence intensity levels of *tnfa:GFP* in larvae in **(G)**. Mean ± SEM, *n* = 36 cells from six embryos representative of three independent experiments. *P*-values shown are: *P < 0.05, **P < 0.01, and ***P < 0.001, two way ANOVA with Bonferonni post-test adjustment.

We next tested whether injury- and infection-induced *tnfa* are Hif-1α and cyclooxygenase dependent processes. Induction of *tnfa:GFP* expression after injury was not blocked by dominant negative Hif-1α (Figures 3A,B), and this was also the case for Mm infection-induced *tnfa:GFP* expression (Figures 3C,D). Similarly, neither genetic nor pharmacological Hif-1α stabilization were additive to the *tnfa:GFP* expression caused by either injury (Figures 3E,F) or Mm infection (Figures 3G,H; (Figures S11A,B). These data indicate that the early *tnfa* response of injury and Mm infection do not require active Hif-1α. Macrophage *tnfa:GFP* expression induced after injury was not abrogated by cyclooxygenase inhibition using NS398 (Figures 3E,F). Similarly, cyclooxygenase inhibition using either SC560 or NS398 did not alter the expression of Mm-induced *tnfa:GFP* (Figures 3G,H). SC560 and NS398 mediated inhibition of Hif-1α-induced *tnfa* expression did not diminish the host-protective effect of DA Hif-1α (Figures S12A–C) nor DMOG treated larvae (Figures S12D–F), suggesting that the presence of Mm induced *tnfa* is sufficient for protection. Our data indicate that Hif-1α-induced macrophage *tnfa* expression via cyclooxygenase differs from DAMP- (injury) or PAMP- (Mm) induced *tnfa* mechanisms.

### Blocking Cyclooxygenase Independent Arachidonic Acid Pathways Do Not Abrogate Hif-1α Upregulation of *tnfa:GFP*

**Figure 4:**
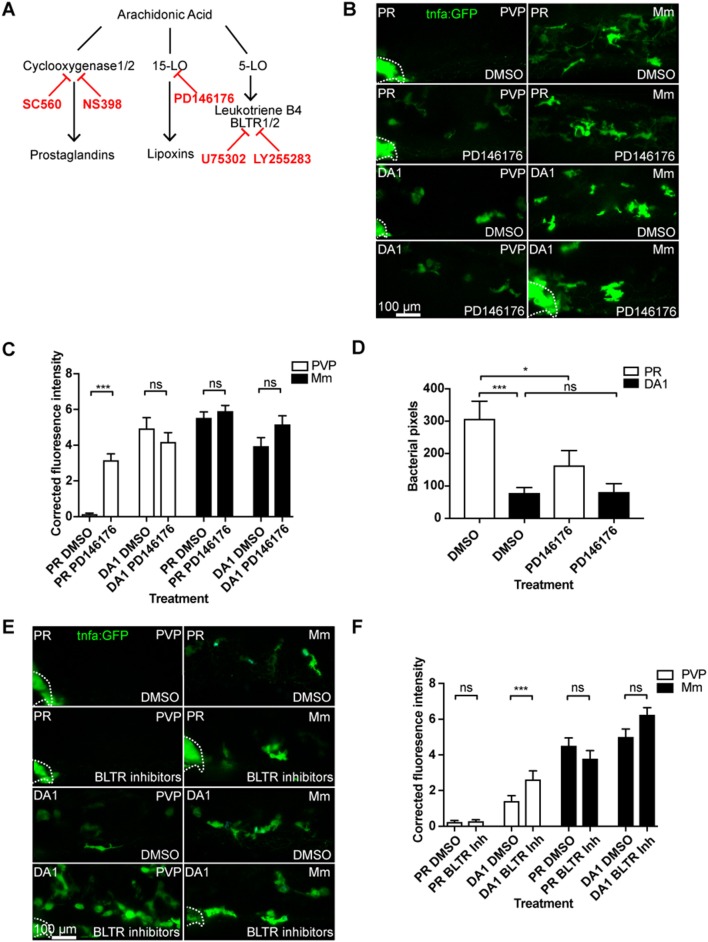
Blocking 15-lipoxygenase or leukotriene B4 receptors does not abrogate DA-Hif-1α-upregulation of *tnfa:GFP*. **(A)** Schematic of the arachidonic pathway indicating where the inhibitors act. **(B)** Confocal micrographs of 1 dpi caudal vein region of Mm mCrimson and PVP infected larvae in the *TgBAC(tnfa:GFP)pd1028* line. Phenol red (PR) and dominant active Hif-1α (DA1) injected larvae were treated with DMSO or PD146176 (15-Lipoxygenase inhibitor). Dotted lines indicate the yolk extension of the larvae where there is non-specific fluorescence. **(C)** Corrected fluorescence intensity levels of *tnfa:GFP* in larvae in **(B)**. Mean ± SEM, *n* = 42 cells from seven embryos accumulated from three independent experiments. *P*-values shown are: *P < 0.05, **P < 0.01, and ***P < 0.001, two way ANOVA with Bonferonni post-test adjustment. **(D)** Bacterial burden at 4 dpi after injection of DA Hif-1α (DA1) and 24 h of the 15-lipoxygenase inhibitor PD146176, using DMSO as a negative solvent control. Data shown are mean ± SEM, *n* = 48–50 as accumulated from three independent experiments. *P*-values shown are: *P < 0.05, **P < 0.01, and ***P < 0.001, two way ANOVA with Bonferonni post-test adjustment. **(E)** Confocal micrographs of 1 dpi caudal vein region of Mm mCrimson and PVP infected larvae in the *TgBAC(tnfa:GFP)pd1028* line. Phenol red (PR) and dominant active Hif-1α (DA1) injected larvae were treated with DMSO or U75302/LY255283 (BLTR1/2 inhibitors). **(F)** Corrected fluorescence intensity levels of *tnfa:GFP* confocal z-stacks in uninfected larvae (PVP, empty bars) and infected larvae (Mm, filled bars) at 1 dpi of data shown in **(E)** after treatment with DMSO or U75302/LY255283 (BLTR1/2 inhibitors). Data shown are mean ± SEM, *n* = 30 cells from five embryos accumulated from two independent experiments. *P*-values shown are: *P < 0.05, **P < 0.01, and ***P < 0.001, two way ANOVA with Bonferonni post-test adjustment.

To investigate whether the effect of the cyclooxygenase inhibitors on *tnfa* was specific to the prostaglandin path of arachidonic acid signaling, we targeted the lipoxin and leukotriene producing arms using the 15-Lipoxygenase inhibitor PD146176 and leukotriene B4 receptor antagonists (Figure 4A) (33, 34). The 15-Lipoxygenase inhibitor PD146176 did not block the *tnfa:GFP* induced by DA Hif-1α (Figures 4B,C). Furthermore, PD147176 increased *tnfa:GFP* expression on its own in the absence of infection, although not to the same extent as DA Hif-α (Figures 4B,C). PD146176 also did not affect *tnfa:GFP* expression after Mm infection (Figures 4B,C), nor did it block the protective effect of DA Hif-1α. Treatment with PD146176 was sufficient to decrease Mm burden, but not to the same extent as DA Hif-1α (Figure 4D). Leukotriene B4 inhibition, using the BLTR1/2 antagonists U75302 and LY255283, did not increase *tnfa:GFP* levels and also did not block DA Hif-1α-induced *tnfa:GFP* (Figures 4E,F). These data indicate that blocking components of the lipoxygenase dependent arms of the arachidonic acid pathway does not block the Hif-1α effect on *tnfa* expression and do not replicate the effects observed by blocking the cyclooxygenase dependent arm.

### Hif-1α-Induced *tnfa:GFP* Requires Active Prostaglandin E2

**Figure 5:**
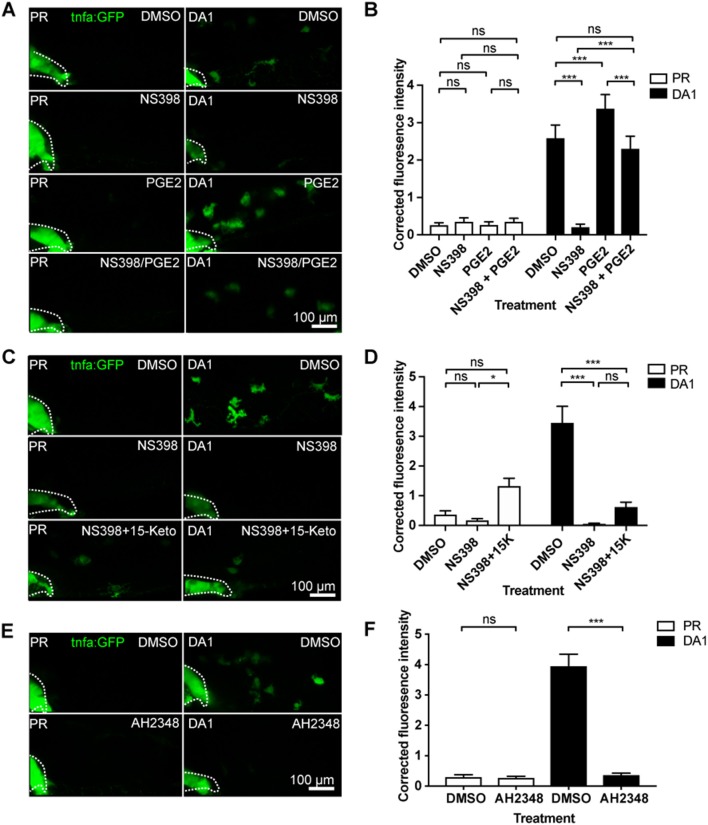
Hif-1α-induced *tnfa:GFP* requires active prostaglandin E2. **(A)** Confocal micrographs of 1 dpi caudal vein region in the *TgBAC(tnfa:GFP)pd1028* line. Phenol red (PR) and dominant active Hif-1α (DA1) injected larvae were treated with DMSO or NS398 (Cox-2 inhibitor) in the presence or absence of endogenous prostaglandin E2 (PGE2). All larvae are PVP injected. **(B)** Corrected fluorescence intensity levels of *tnfa:GFP* in larvae in **(A)**. Mean ± SEM, *n* = 54 cells from nine embryos accumulated from three independent experiments. *P*-values shown are: *P < 0.05, **P < 0.01, and ***P < 0.001, two way ANOVA with Bonferonni post-test adjustment. **(C)** Confocal micrographs of 1 dpi caudal vein region in the *TgBAC(tnfa:GFP)pd1028* transgenic line. Phenol red (PR) and dominant active Hif-1α (DA1) injected larvae were treated with DMSO or NS398 (Cox-2 inhibitor) in the presence or absence of endogenous 15-keto prostaglandin E2 (15K). All larvae are PVP injected. *P*-values shown are: *P < 0.05, **P < 0.01, and ***P < 0.001, two way ANOVA with Bonferonni post-test adjustment. **(D)** Corrected fluorescence intensity levels of *tnfa:GFP* in larvae in **(C)**. Mean ± SEM, *n* = 24 cells from four embryos accumulated from two independent experiments. **(E)** Confocal micrographs of 1 dpi caudal vein region in the *TgBAC(tnfa:GFP)pd1028* transgenic line. Phenol red (PR) and dominant active Hif-1α (DA1) injected larvae were treated with DMSO or AH2348 (EP4 inhibitor). All larvae are PVP injected. **(F)** Corrected fluorescence intensity levels of *tnfa:GFP* in larvae in **(E)**. Mean ± SEM, *n* = 72 cells from 12 embryos accumulated from three independent experiments. *P*-values shown are: *P < 0.05, **P < 0.01, and ***P < 0.001, two way ANOVA with Bonferonni post-test adjustment.

A key family of immune regulators downstream of arachidonic acid and cyclooxygenases are prostaglandins. The best characterized of these as a regulator of macrophage function is prostaglandin E2 (PGE2) (41). We tested whether PGE2 was a mediator in the HIF/COX/TNF pathway by addition of exogenous PGE2 to DA Hif-1α larvae (35). Exogenous PGE2 had no effect on *tnfa:GFP* expression in the absence of infection (Figures 5A,B). However, PGE2 was able to rescue the decrease in *tnfa:GFP* expression after inhibition of either Cox-1 (Figure S13) or Cox-2 (Figures 5A,B) in DA Hif-1α larvae. Furthermore, addition of PGE2 alone was sufficient to increase the DA Hif-1α-induced *tnfa:GFP* expression (Figures 4A,B). These *tnfa:GFP* activating effects were not observed by addition of exogenous 15-keto prostaglandin E2, an immunologically inactive degradation product of PGE2 (Figures 5C,D) (35, 42, 43). These data indicate that Hif-1α-induced *tnfa:GFP* requires active prostaglandin E2.

PGE2 signals via prostanoid receptors. In human and zebrafish there are four different receptors, EP1-4 (44). Of these, EP4 is the best characterized in immune cells and is expressed on human macrophages. When EP4 is blocked, macrophage pro-inflammatory cytokine release is decreased (45). We therefore tested a previously published EP4 antagonist AH23848 (36). We first demonstrated that AH23848 did not activate Hif-1α transcription using the *Tg(phd3:GFP)* hypoxia reporter line (Figure S6). The increased level of *phd3:GFP* induced by hydroxylase inhibitors was also not altered by AH23484 treatment, demonstrating that Hif-1α transcriptional potential is not affected by EP4 inhibition (Figure S8). Addition of AH23848 abrogated dominant active Hif-1α-induced *tnfa:GFP*, indicating that Hif-1α stabilization acts through PGE2 signaling via EP4 to upregulate Tnfa expression (Figures 5E,F).

## Discussion

Control of macrophage function during homeostasis and disease are critical for maintaining healthy tissues and must integrate changes in the local microenvironment with cytokine signaling. Understanding of the signaling pathways that link microenvironment with macrophage phenotypic outcomes may identify novel therapeutic avenues for control of macrophages during disease.

Here, we show that a disease relevant microenvironmental cue, hypoxia signaling via Hif-1α, upregulates macrophage *tnfa* expression in a cyclooxygenase dependent. TNF upregulation by hypoxia has been previously demonstrated in a range of mammalian cells, both directly, through HIF responsive elements (HREs) in its promotor region, and indirectly (46–49). Here, we addressed hypoxia-induced *tnfa* levels using promoter driven GFP expression in two zebrafish transgenic lines as a proxy for endogenous *tnfa* expression (18, 20). Changes in endogenous *tnfa* expression on a wholebody-level after Hif-1α stabilization were not observable by qPCR (data not shown), presumably because the changes in transcription at the macrophage level are too small to be detected using these methods due to macrophages representing a small proportion of the zebrafish larvae, a challenge that was previously reported in wholebody detection of another pro-inflammatory cytokine, *il-1*β (37). These promoter driven *tnfa* transgenic lines have therefore opened up opportunities to study *tnfa* induction *in situ* in the *in vivo* zebrafish. We observed increased *tnfa* expression with stabilized Hif-1α, demonstrating that hypoxia signaling alone can lead to a change of macrophage phenotype, consistent with our previous observations with *il-1*β expression, suggestive of a pro-inflammatory response (37). Our findings are also in line with HIF-1α stabilization being important for the production of TNF and other pro-inflammatory molecules, such as nitric oxide (NO), leading to host-protective effects against group A *Streptococcus* in *in vivo* murine models (50). Not only could *tnfa* activation be achieved by genetic stabilization of Hif-1α, but also using hypoxia mimetics (hydroxylase inhibitors) and physiological hypoxia, that stabilize endogenous Hif-1α. These findings indicate that the Hif-1α-Tnfa axis is targetable by pharmaceuticals and could be druggable during disease.

A similar *tnfa* induction was induced by infection and inflammation in our *in vivo* zebrafish models. It has been widely demonstrated that Tnfa is required for control of early Mm infection, with perturbation of Tnfa signaling leading to high infectious burdens (51). While Hif-1α-induced *tnfa* was downregulated by cyclooxygenase inhibition, it was notable that the high level of *tnfa* expression after inflammation or infection was not affected by cyclooxygenase inhibition. These findings are consistent with the novel HIF/COX/TNF macrophage axis being independent of infection/inflammation-induced macrophage Tnfa. Mm-induced Tnfa is widely reported as being toll-like receptor (TLR) mediated, and our data indicate that Mm-induced levels of Tnfa are sufficient for control of bacteria in the presence of stabilized Hif-1α (28, 52). In the absence of the TLR adapter protein MyD88, bacterial induction of Tnfa is lost, demonstrating the central role of TLR pathways in post-infection Tnfa induction (28, 52–54). The best characterized exogenous TLR signal, LPS via TLR4, can induce Tnfa production via activation of the central pro-inflammatory regulator NFκB (54, 55). As cyclooxygenase inhibition did not alter either inflammation or infection driven *tnfa* it suggests that these complex damage/infection driven mechanisms of Tnfa induction are not dependent on the HIF/COX/TNF pathway. Our data demonstrate that Hif-1α stabilization can stimulate a macrophage *tnfa* producing response in the absence of a TLR stimulus, which may have important implications in sterile diseased tissue and infers an alternative macrophage tuning mechanism.

Hypoxic regulation of TNF via COX has previously been demonstrated in mammalian osteoblasts, however, little is known about this interaction (14). Here, we demonstrate that Hif-1α upregulation of macrophage *tnfa* is likely to be via the production of PGE2 signaling via the EP4 receptor. PGE2 is an inflammatory mediator that is known to affect macrophage polarization states during infection, and synergises with cytokines to amplify pro-inflammatory responses (41, 56). The degradation metabolite 15-keto-PGE2 did not rescue the loss of *tnfa* expression after cyclooxygenase inhibition, consistent with previous reports that 15-keto-PGE2 is unable to bind the prostaglandin E2 EP receptors, thus demonstrating a requirement for active PGE2 (57).

Here, we demonstrate that a macrophage HIF/COX/TNF pathway is found in human monocyte derived macrophages, indicating that this pathway may have conserved roles in macrophage homeostasis and disease, and opening up possibilities for pharmaceutical intervention to promote proinflammatory macrophage phenotypes in human disease. Our findings in human MDMs indicate that the HIF/COX/TNF pathway may, at least in part, be cell-autonomous to macrophages, however, this was not tested *in vivo* where it is likely that contributing signals from other celltypes, for example endothelial cells, may play important roles in macrophage phenotype outcomes (58).

Although the effect of the HIF/COX/TNF axis in early Mm infection was negligible, this pathway is likely to have important roles in other disease situations. Mycobacterial infection, along with many other bacterial pathogens, stimulates early TNF production, however some pathogens remain undetected by macrophages during early disease. An example of this is in Lyme disease, caused by *Lyme borreliosis*, where early low TNF production is associated with worse disease outcomes (59). Multiple infectious/inflammatory disease pathologies have hypoxia as a feature of the tissue microenvironment, concurrent with the presence of macrophages. For example, Hif-1α mediated TNF activation may be pertinent in later TB infection situations where granulomas have stabilized Hif-1α due to necrotic and hypoxic centers (60, 61). Hypoxia is a key hallmark of cancers with high levels of HIF-1α widely found in those that produce large tumors where the center is hypoxic (62). These tumors can also produce cyclooxygenase/PGE2, where these important immunomodulatory signals are required for cellular adaption to the tumor microenvironment (63). Macrophages play central roles in cancer-inflammation that could be exploited utilizing pharmaceutical control of this novel HIF/COX/TNF mechanism. Further investigation of the HIF/COX/TNF axis in models where hypoxia is a key hallmark of disease pathology is required to uncover the full therapeutic relevance of this potentially important novel macrophage pathway.

In conclusion, we have identified a novel mechanism for macrophage *tnfa* production via Hif-1α and cyclooxygenase that is found in zebrafish and humans. Importantly, this axis links a commonly found microenvironmental cue, hypoxia, to a macrophage cytokine, TNF. We provide strong evidence to show that this response acts via the cyclooxygenase/PGE2 arm of the arachidonic pathway. Due to the central roles of these modulators in disease microenvironments we anticipate that this HIF/COX/TNF pathway may have important implications in conditions such as inflammation, infection and cancer.

## Data Availability Statement

The datasets generated for this study are available on request to the corresponding author.

## Ethics Statement

The studies involving human participants were reviewed and approved by the South Sheffield Research Ethics Committee (07/Q2305/7), University of Sheffield, Firth Court, Western Bank, S10 2TN. The healthy participants provided their written informed consent to participate in this study. Animal work was performed following UK law: Animal (Scientific Procedures) Act 1986, under Project License P1A4A7A5E. Ethical approval was granted by the University of Sheffield Local Ethical Review Panel, University of Sheffield, Firth Court, Western Bank, Sheffield, S10 2TN.

## Author Contributions

PE and AL conceived and designed the experiments, performed the experiments, and analyzed the data. PE wrote the paper.

### Conflict of Interest

The authors declare that the research was conducted in the absence of any commercial or financial relationships that could be construed as a potential conflict of interest.

## Abbreviations

LOX: lipoxygenase
BLTR: leukotriene receptor
COX: cyclooxygenase
DAMP: damage associated molecular pattern
DMOG: dimethyloxaloylglycine
DMSO: dimethyl sulfoxide
Dpf: day post-fertilization
Dpi: day post-infection
FBS: fetal bovine serum
GFP: green fluorescent protein
HEPES: 4-(2-hydroxyethyl)-1-piperazineethanesulfonic acid
HIF: hypoxia inducible factor
hMDM: human monocyte derived macrophage
Hpf: hour post-fertilization
Hpi: hour post-infection
Hpw: hour post-wound
HRE: HIF responsive element
IL: Interleukin
Mm: *Mycobacterium marinum*
NFκB: nuclear factor kappa-light-chain-enhancer of activated B cells
PAMP: pathogen associated molecular pattern
PGE2: Prostaglandin E2
PMBC: peripheral blood mononuclear cell
TLR: toll-like receptor
TNF: tumor necrosis factor.
