# Supplementary material for: Hypoxia Induces Macrophage tnfa Expression via Cyclooxygenase and Prostaglandin E2 in vivo
Source: Front Immunol. 2019 Sep 27;10:2321. doi: 10.3389/fimmu.2019.02321 (PMC6776637; doi:10.3389/fimmu.2019.02321)
Supplement: Supplementary file 1 [file Data_Sheet_1.pdf]

Supplementary Figure Legends

Figure S1

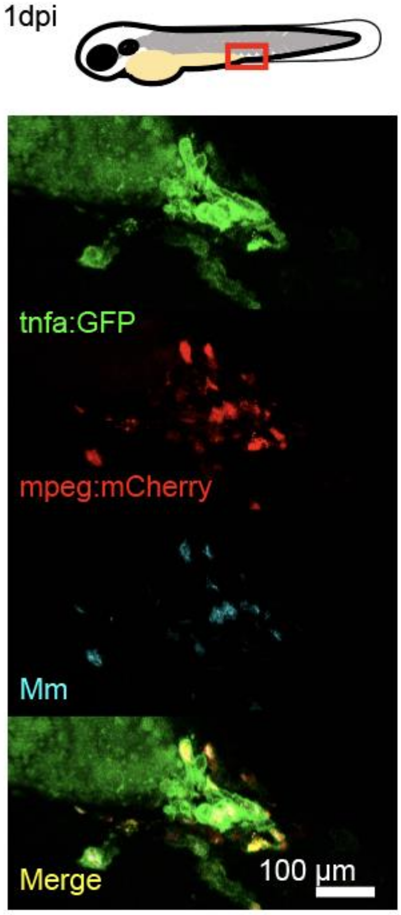

Figure S1. Mm infection induced *tnfa:GFP* in macrophages in a second transgenic line.

Fluorescent confocal micrographs of 1dpi caudal vein region of infection. *tnfa* expression was detected by GFP levels, in green, using the *Tg(tnfa:eGFP-F)ump5Tg* transgenic line. Macrophages are shown in red using a *Tg(mpeg1:mCherry-F)ump2Tg* line. Mm Crimson is shown in the blue channel. Dotted lines indicate the yolk extension of the larvae where there is non-specific fluorescence.

Figure S2

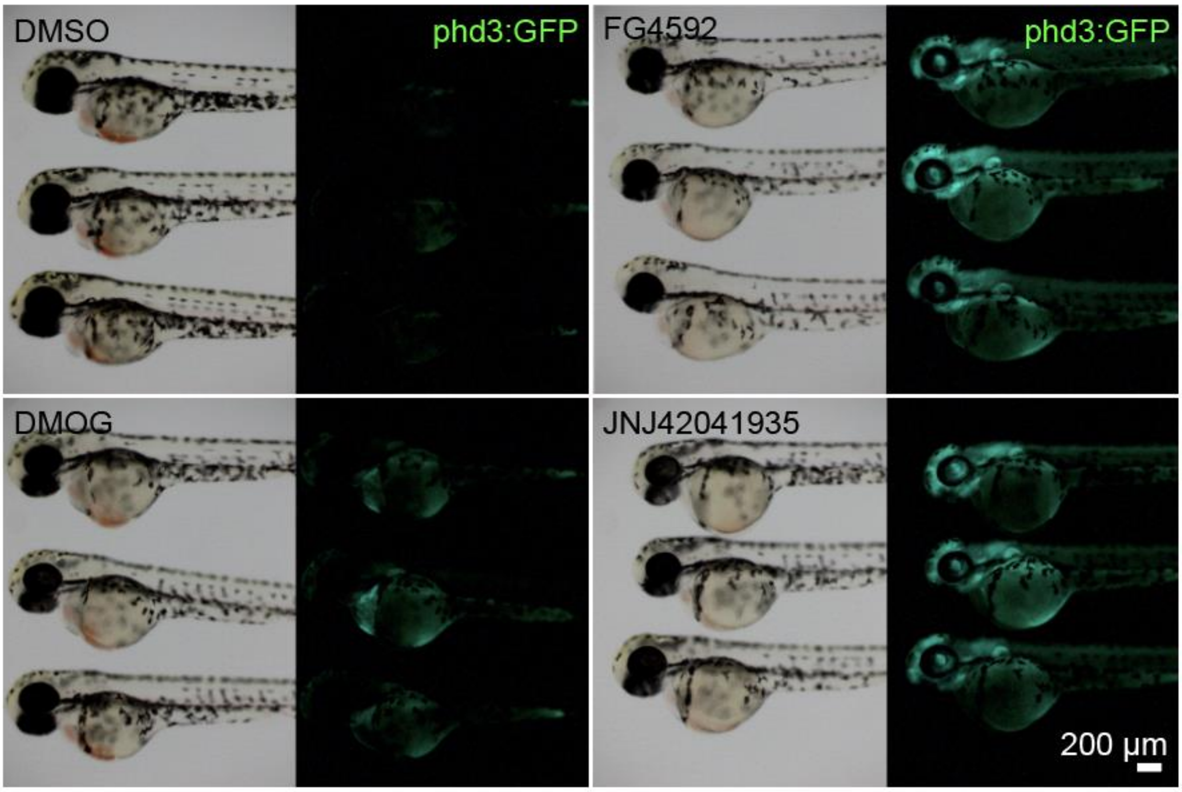

Figure S2. Hydroxylase inhibitors induce expression of the *phd3:GFP* hypoxia reporter transgene.

Fluorescent micrographs of 48hpf *Tg(phd3:eGFP)i144* hypoxia reporter zebrafish after treatment with DMSO, DMOG, FG4592 or JNJ42041935 from 32-48hpf. Images are representative of 3 independent experiments each with 20 fish per group.

Figure S3

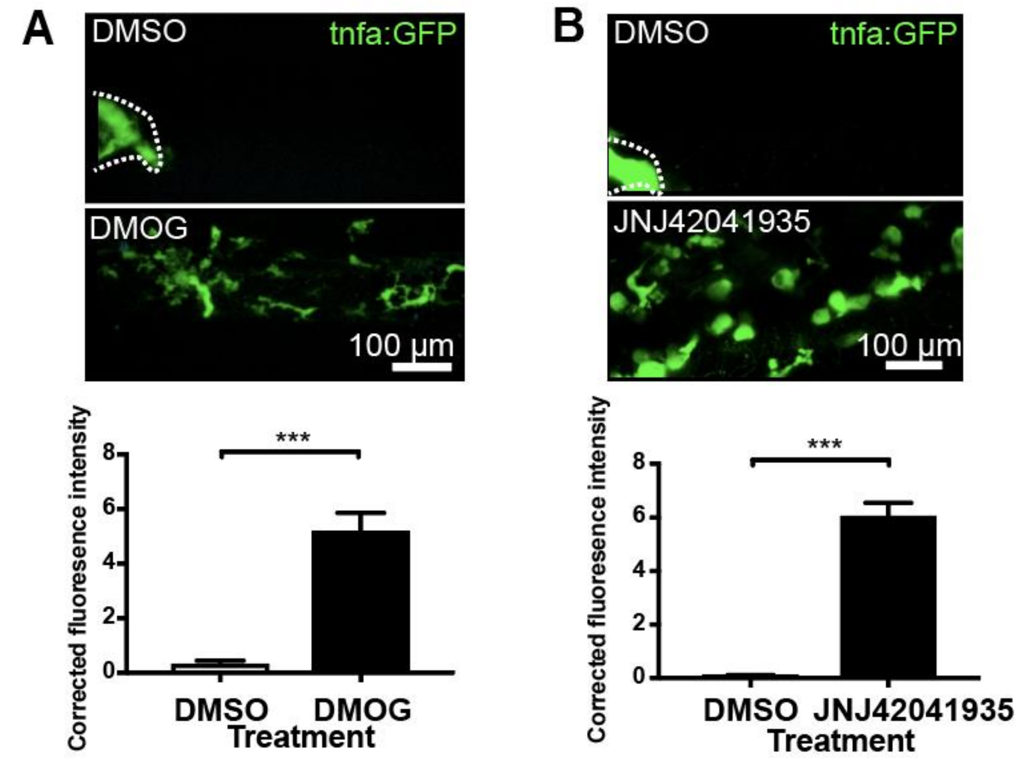

Figure S3. Hydroxylase inhibitors DMOG and JNJ-42041935 increase *tnfa:GFP* expression

(A) Confocal micrographs of 2dpf *TgBAC(tnfa:GFP)pd1028* larvae treated with DMOG or DMSO control. Graph shows corrected fluorescence intensity levels of *tnfa:GFP.* Mean ± SEM, n=24 cells from 6 embryos representative of 2 independent experiments.

(B) Confocal micrographs of 2dpf *TgBAC(tnfa:GFP)pd1028* larvae treated with the PHD inhibitor JNJ-42041935 or a DMSO solvent control. Graph shows corrected fluorescence intensity levels of *tnf-a:GFP*. Mean ± SEM, n=48 cells from 8 embryos accumulated from 2 independent experiments.

Figure S4

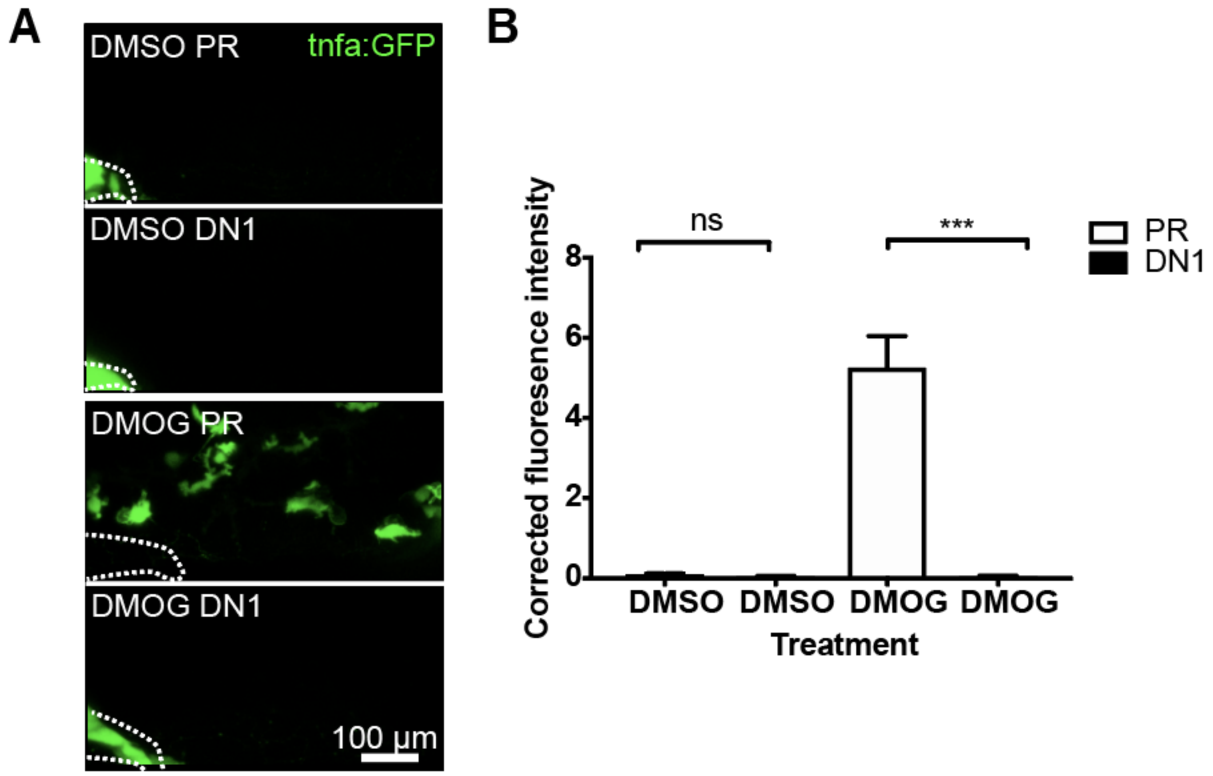

Figure S4. DMOG induced tnfa:GFP is blocked by dominant negative Hif-1α

(A) Confocal micrographs of 2dpf *TgBAC(tnfa:GFP)pd1028* larvae treated with the PHD inhibitor FG4592 or a DMSO solvent control. Larvae were injected at the 1 cell stage with dominant negative (DN) Hif-1α or phenol red (PR) control.

(B) Corrected fluorescence intensity levels of *tnfa:GFP* in larvae in (H). Mean ± SEM, n=24 cells from 4 embryos accumulated from 2 independent experiments.

Figure S5

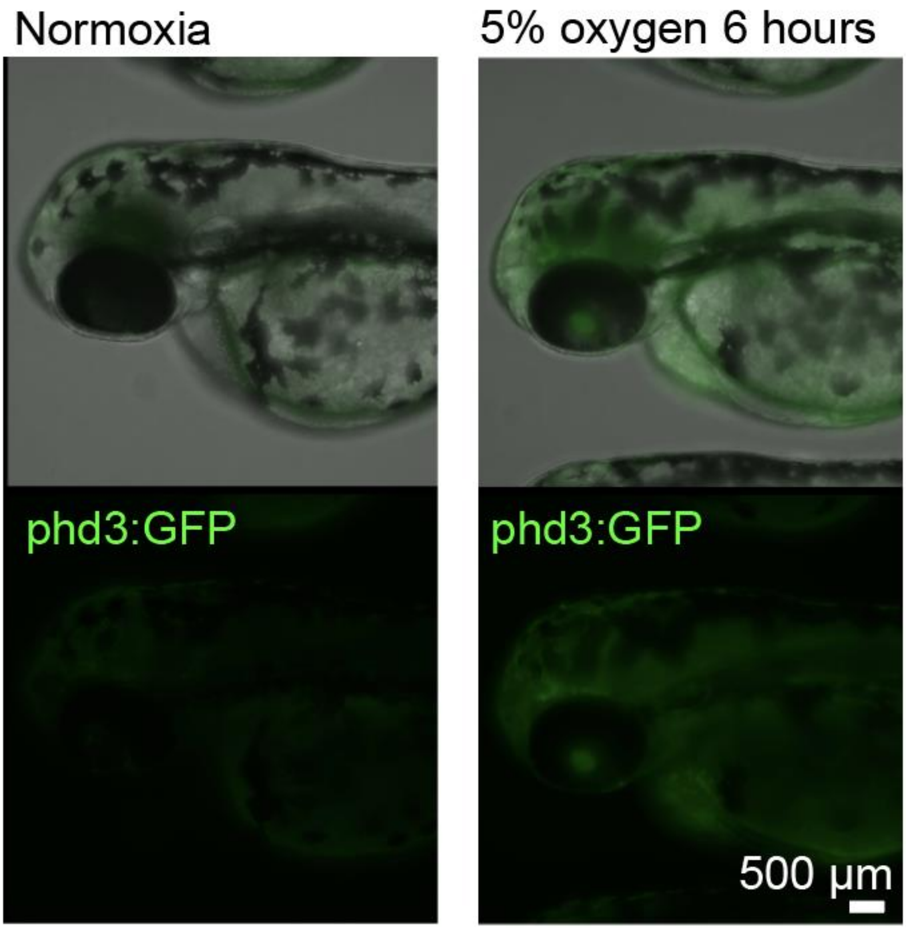

Figure S5. 5% oxygen induces expression of the *phd3:GFP* hypoxia transgene.

Fluorescent micrographs of 48hpf *Tg(phd3:eGFP)i144* hypoxia reporter zebrafish after incubated in 6 hours of 5% oxygen from 32-38hpf, or normoxic controls. Images are representative of 3 independent experiments each with 20 fish per group.

Figure S6

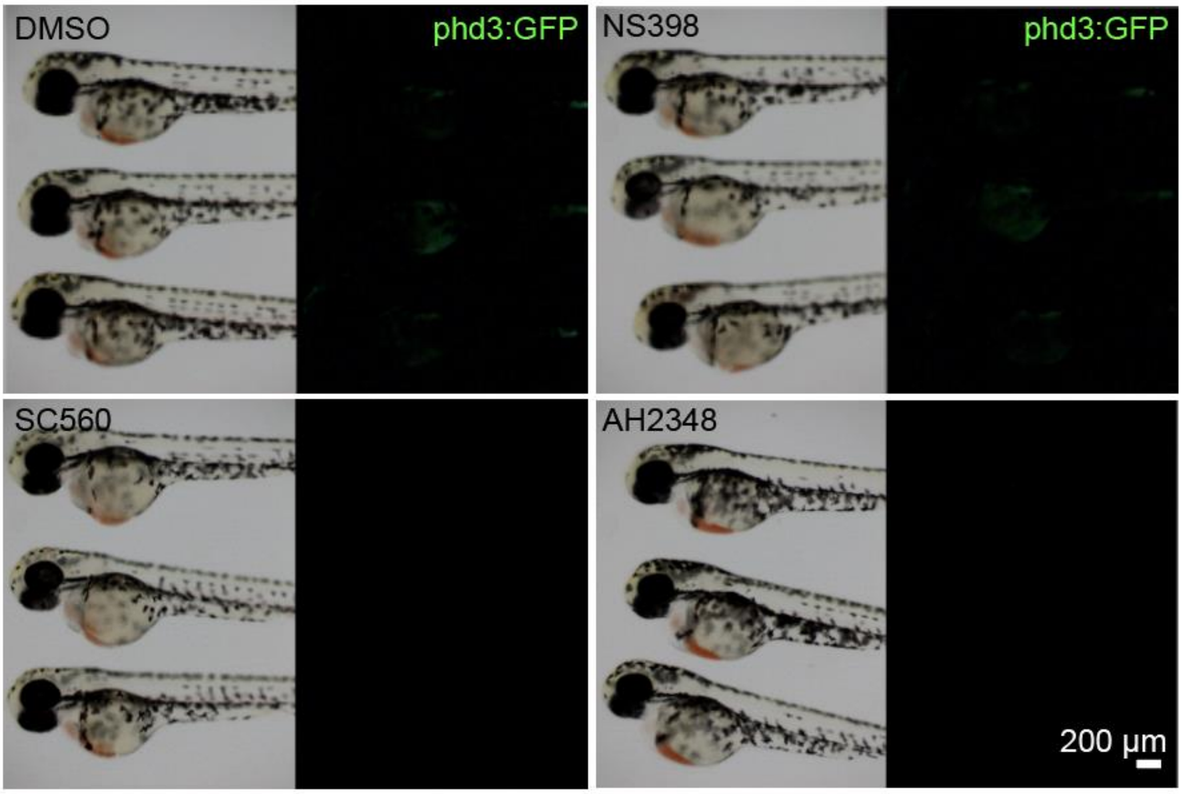

Figure S6. Arachidonic acid component pathway inhibitors do not induce expression of the *phd3:GFP* hypoxia transgene.

Fluorescent micrographs of 48hpf *Tg(phd3:eGFP)i144* hypoxia reporter zebrafish after treatment with inhibitors of the arachidonic acid pathway, DMSO, SC560, NS398 and AH2348 from 32-48hpf. Images are representative of 3 independent experiments each with 20 fish per group.

Figure S7

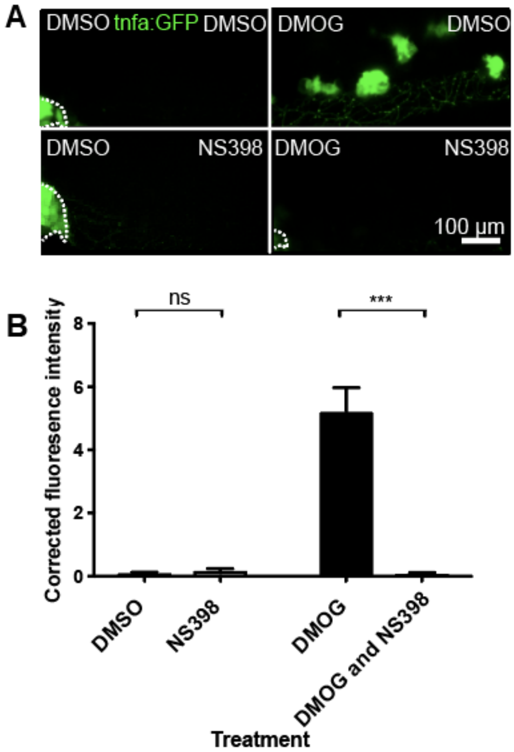

Figure S7: DMOG induced *tnfa:GFP* is abrogated by Cox-2 inhibition by NS398

(A) Confocal micrographs of 2dpf caudal vein region of larvae in the *TgBAC(tnfa:GFP)pd1028* line. DMSO and DMOG treated larvae were co-treated with DMSO and NS398 (Cox-2 inhibitor). Dotted lines indicate the yolk extension of the larvae where there is non-specific fluorescence.

(B) Corrected fluorescence intensity levels of *tnfa:GFP* in larvae in (A). Mean ± SEM, n=24 cells from 4 embryos representative of 2 independent experiments.

Figure S8

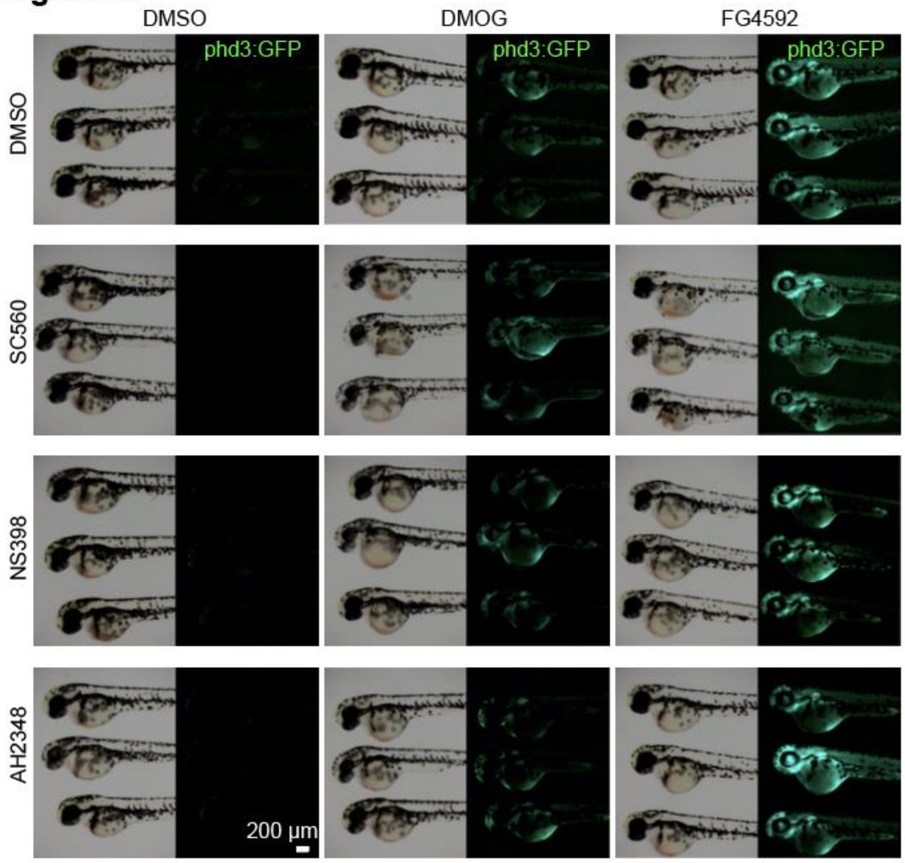

Figure S8. Arachidonic acid pathway inhibitors do not block hydroxylase inhibitors inducing transcription of the *phd3:GFP* hypoxia transgene.

Fluorescent micrographs of 48hpf *Tg(phd3:eGFP)i144* hypoxia reporter zebrafish after treatment with hypoxia mimetics, DMSO, DMOG, and FG4592, co-treated with inhibitors of arachidonic acid pathway components, DMSO, SC560, NS398 and AH2348 from 32-48hpf. Images are representative of 3 independent experiments each with 20 fish per group.

Figure S9

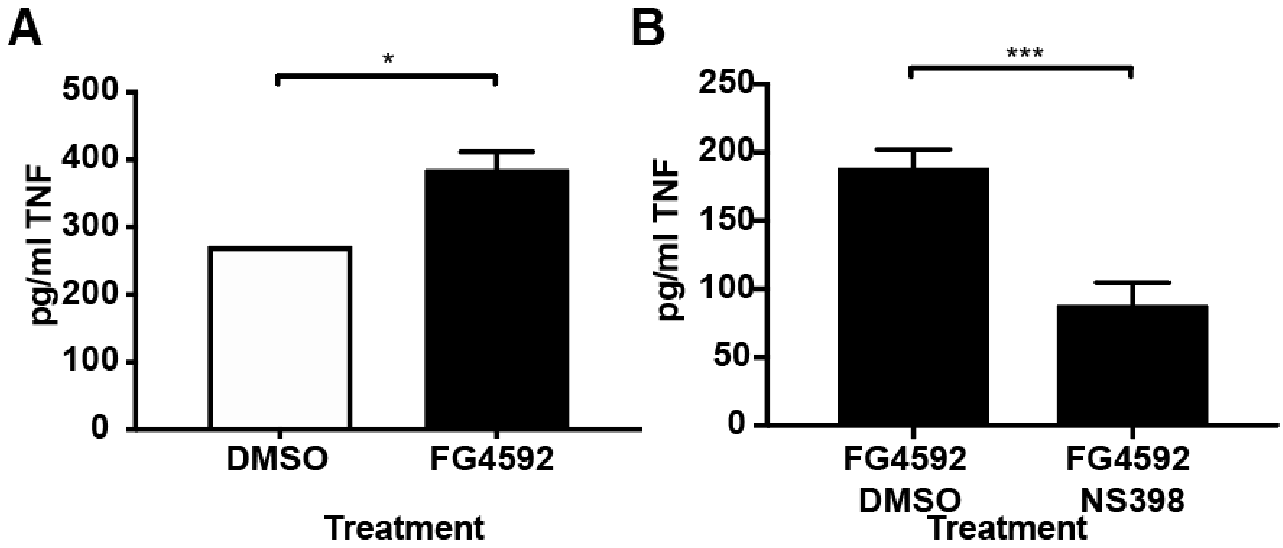

Figure S9. FG4592 induces TNF in human MDMs in a COX-2 dependent manner

(A) TNF ELISA of human monocyte derived macrophages treated with LPS and incubated in normoxia treated with DMSO or FG4592. LPS negative controls were performed but TNF produced in these groups was below detectable levels. Mean ± SEM, n=2 biological repeats from 2 donors. Error bar in DMSO group is less than 1 and too small to be observed.

(B) TNF ELISA of human monocyte derived macrophages treated with LPS and incubated in normoxia treated with FG4592 with or without treatment with NS398. LPS negative controls were performed but TNF produced in these groups was below detectable levels. Mean ± SEM, n=6 biological repeats from 3 donors.

Figure S10

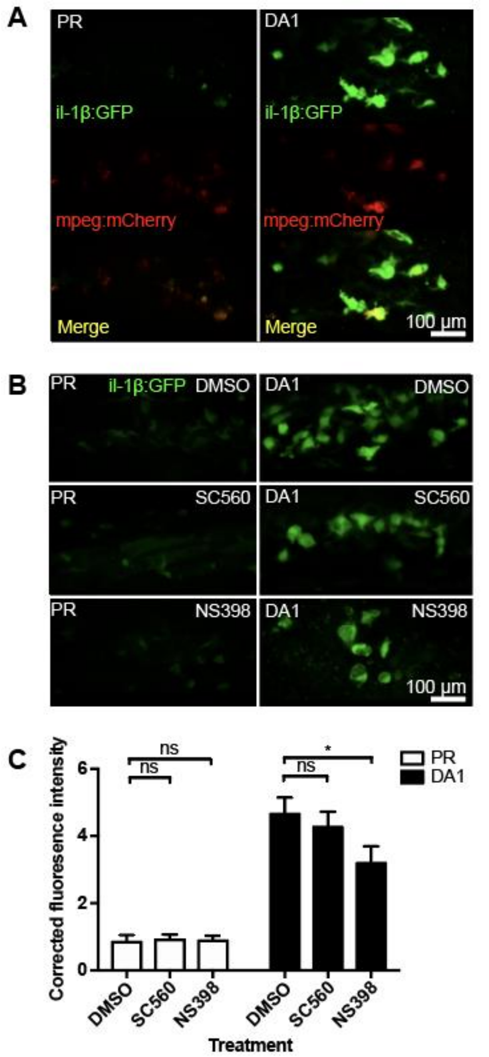

Figure S10. DA-Hif-1α induced *il-1β:GFP* is not altered by Cox-1 inhibition and decreased by Cox-2 inhibition.

(A) Fluorescent confocal micrographs of 2dpf caudal vein region. *il-1β* expression was detected by GFP levels, in green, using the *TgBAC(il-1β:GFP)SH445* transgenic line. Macrophages are shown in red using a *Tg(mpeg1:mCherryCAAX)sh378* line. Without infection there is little detectable *il-1β:GFP* expression in phenol red (PR) controls, while DA-Hif-1α macrophages have higher levels of *il-1β:GFP* in the Mm group.

(B) Fluorescent confocal micrographs of 1dpi caudal vein region of PVP injected larvae. *il-1β* expression was detected by GFP levels. Phenol red (PR) and dominant active Hif-1α (DA1) injected larvae were treated with DMSO, SC560 (COX-1 inhibitor) and NS398 (COX-2 inhibitor).

(C) Corrected fluorescence intensity levels of *il-1β:GFP* confocal z-stacks in PVP injected larvae at 1dpi from (B). Data shown are mean ± SEM, n=36 cells from 6 embryos representative of 3

independent experiments.

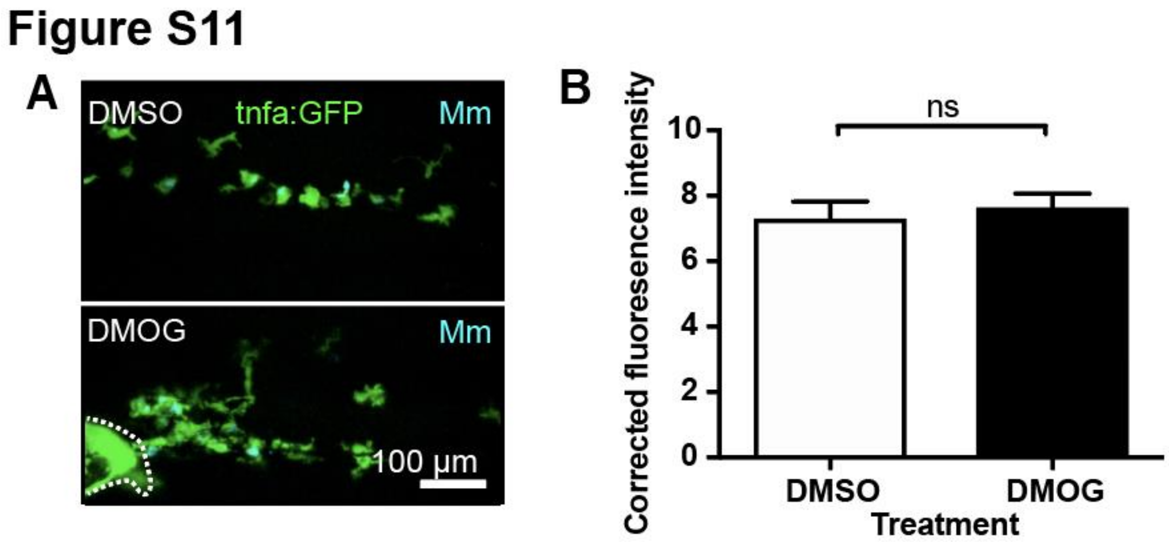

Figure S11. Mm infection induced *tnfa:GFP* is not altered by DMOG induced Hif-1α stabilisation

(A) Fluorescent confocal micrographs of 2dpf Mm infected *TgBAC(tnfa:GFP)pd1028* transgenic larvae treated with DMOG or DMSO control. Dotted lines indicate the yolk extension of the larvae where there is non-specific fluorescence.

(B) Graph shows corrected fluorescence intensity levels of *tnfa:GFP* confocal z-stacks in larvae. DMOG treated larvae (filled bars) had significantly increased *tnfa:GFP* levels compared to DMSO controls (white bars). Data shown are mean ± SEM, n=24 cells from 6 embryos representative of 2 independent experiments.

Figure S12

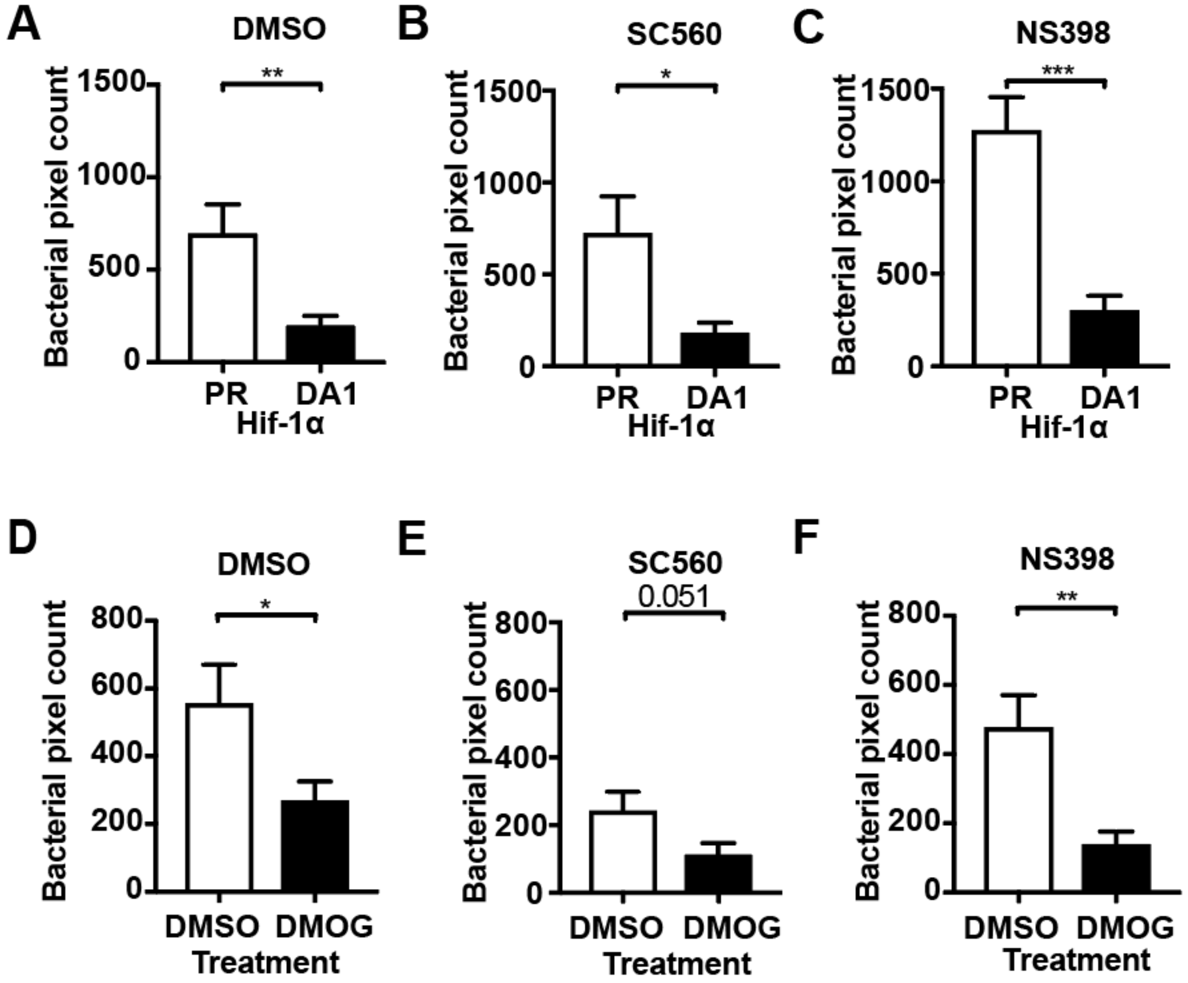

Figure S12: Cyclooxygenase inhibition does not block the protective effect of Hif-1α stabilisation in Mm infection.

(A) Bacterial burden of DMSO treated larvae at 4dpi after injection with phenol red (PR) or dominant active Hif-1α (DA1). Data shown are mean ± SEM, n=35 as accumulated from 3 independent experiments.

(B) Bacterial burden of SC560 treated larvae at 4dpi after injection with phenol red (PR) or dominant active Hif-1α (DA1). Data shown are mean ± SEM, n=35 as accumulated from 3 independent experiments.

(C) Bacterial burden of NS398 treated larvae at 4dpi after injection with phenol red (PR) or dominant active Hif-1α (DA1). Data shown are mean ± SEM, n=35 as accumulated from 3 independent experiments.

(D) Bacterial burden of DMSO treated larvae at 4dpi after co-treatment with DMSO or DMOG. Data

shown are mean ± SEM, n=33-36 as accumulated from 3 independent experiments.

(E) Bacterial burden of SC560 treated larvae at 4dpi after co-treatment with DMSO or DMOG. Data shown are mean ± SEM, n=33 as accumulated from 3 independent experiments.

(F) Bacterial burden of NS398 treated larvae at 4dpi after co-treatment with DMSO or DMOG. Data shown are mean ± SEM, n=33 as accumulated from 3 independent experiments.

Figure S13

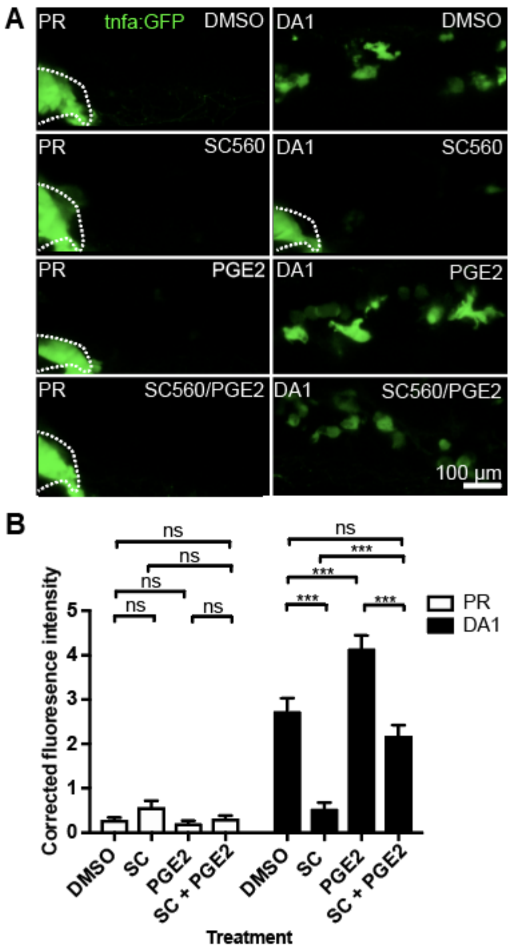

Figure S13. Hif-1α-induced *tnfa:GFP* requires active prostaglandin E2

(A) Confocal micrographs of 1dpi caudal vein region in the *TgBAC(tnfa:GFP)pd1028* line. Phenol red (PR) and dominant active Hif-1α (DA1) injected larvae were treated with DMSO or SC560 (Cox-1 inhibitor) in the presence or absence of endogenous prostaglandin E2 (PGE2). All larvae are PVP injected. Dotted lines indicate the yolk extension of the larvae where there is non-specific fluorescence.

(B) Corrected fluorescence intensity levels of *tnfa:GFP* in larvae in (A). Mean ± SEM, n=54 cells from 9 embryos accumulated from 3 independent experiments.
